# Concordance in mSEGA Tool to Frailty Diagnosis between Medical Doctors and Nurses

**DOI:** 10.3390/medicines8110063

**Published:** 2021-10-29

**Authors:** Abrar-Ahmad Zulfiqar, Ibrahima Amadou Dembélé

**Affiliations:** Department of Internal Medicine, University Hospital of Strasbourg, 67000 Strasbourg, France; ibrahimaadembele@gmail.com

**Keywords:** frailty, mSEGA grid, elderly subjects, emergency department, concordance study

## Abstract

**Introduction:** It is currently considered that screening for frailty in elderly subjects is a major public health issue. **Methods:** a cross-sectional pilot study involving elderly subjects (over 75 years of age) admitted at the emergency department of the hospital of Troyes, France in the period from 24 August to 30 August 2017 was conducted. The patients were screened for frailty using the modified SEGA (Short Emergency Geriatric Assessment) (part A) grid (mSEGA), correlated with the subjective opinion of the triage nurse and the senior physician. **Results:** 100 patients were included during the pilot study period, the mean age was 84.34 years (range: 75–97), 56 patients (56%) were female, and the average CHARLSON score was 4.28 (range: 0–11). The patients’ previous medical histories were remarkable for cardiovascular diseases. The main reason for hospital admission was fall (26 subjects, 26%). Hospitalization was required for 52 subjects (52%). The average mSEGA score was 6.3 +/− 3.59. The completion time for the SEGAm (part A) score was about 5 minutes. According to Cohen’s kappa, the concordance between the subjective opinion of the triage nurse and the mSEGA grid was average, while the concordance between the subjective opinions of the senior physicians was good. **Conclusion:** The mSEGA score appears to be well-suited and useful in the emergency department. It is easy to use, allows an overall evaluation of the patient, and is not time-consuming.

## 1. Introduction

The elderly population continues to grow, with demographic projections showing the same trend. Indeed, various INSEE reports suggest a 50% increase in the number of dependent elderly persons between 2000 and 2040, reaching 10 million [1]. Emergency departments are, therefore, on the front lines of this demographic shift. They are, in fact, the first step towards hospital admission in elderly subjects. In France, hospital-based health care includes a variety of roles, but emergency physicians are, most of the time, the first medical personnel to evaluate patients. Emergency doctors are the first source of information for other medical and surgical specialists, including geriatricians. The elderly population is very heterogeneous, ranging from strong elderly subjects to dependent elderly ones. Between those two extremes are those subjects considered as “frail”. This concept of frailty is complex due to the fact that each individual reacts differently to the stress factors to which he or she is subjected. Even though there is no general consensus on a definition, it is generally understood that frailty exposes elderly subjects to negative health situations (unplanned hospitalization, institutionalization, excess morbidity, mortality, etc.). Frailty as a clinical syndrome was defined in 2011 by the Société Française de Gérontologie et de la Gériatrie as a decline in the physical reserves across multiple systems, subjecting an elderly person under stress to adverse effects. Its clinical expression is affected by comorbidities and by psychological, social, economic, and behavioral factors [2]. All the authors agree that this is a major morbidity and mortality factor [3]. Some of these factors are reversible, making their detection and prevention a major public health issue. The early detection of these potentially reversible factors through multidisciplinary management seems to be a real public health issue, with the aim of reducing certain avoidable dependencies of aging and, therefore, expenses [4].

In fact, the SAFES study, carried out by a team from Reims, made it possible to highlight an increase in the length of hospitalization in pre-fragile and fragile subjects, as well as a higher rate of re-hospitalization, a significant risk of admission to the hospital, and death [5]. Another study also showed that preventing frailty could delay deaths in 3–5% [6].

Thus, emergency physicians are on the front line of health care in elderly subjects regardless of the reason of admission. The scientific literature provides emergency physicians with scores aiming at screening frailty in elderly subjects, such as the SEGA (Short Emergency Geriatric Assessment) scale, created by a Belgian team [7]. This frailty scale was initially developed for elderly subjects admitted to the emergency department [7] before being modified and validated for use in community-dwelling subjects [8,9].

The aim of our study was to determine the concordance between the subjective evaluation of the nursing triage team and the senior physician regarding the frailty status of an elderly patient in the emergency department through, and the medical use of, the SEGAm grid (part A).

## 2. Patients and Methods

### 2.1. Overview and Study Population

This is a cross-sectional pilot study conducted in the emergency department of the hospital of Troyes (Aube), France. The exploratory investigation was conducted from 24 August 2017 to 30 August 2017. The inclusion criterion was age 75 or more at the time of admission to the emergency department. We enrolled all patients consecutively admitted to the ED across a daily time period (9 a.m.–6 p.m.) during the 7-day enrolment timeframe, the triage nurse being present at this time in the emergency department of Troyes. Patients receiving invasive medical treatments (non-invasive ventilation, intubation), with a life-threatening condition, labeled as receiving palliative or end-of-life care, or coming from nursing homes were excluded.

### 2.2. Data Collected

**Figure 1:**
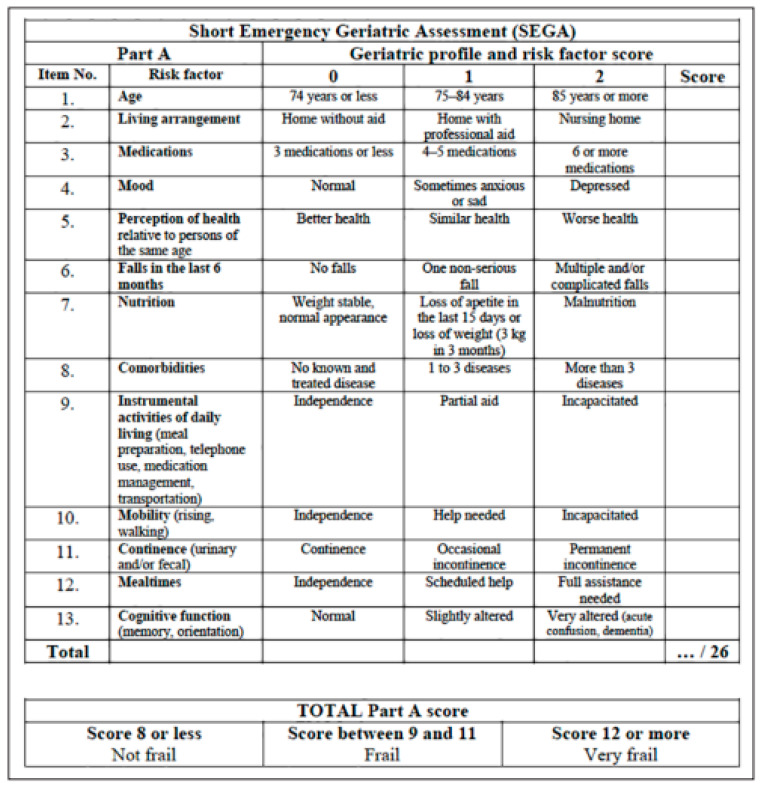
Modified SEGA scale part A.

General variables, such as sex, age, previous medical and surgical history, as well as Charlson Comorbidity Index score, medication, frailty level evaluated through mSEGA grid (part A), and the subjective evaluation of frailty by the triage nurse and the senior physician, were collected. The frailty status of the patient was evaluated using the Short Emergency Geriatric Assessment (SEGA) by an external figure. For the mSEGA (part A) score, the patients were assigned to two groups: non-frail patients (score < 8), and frail or very frail patients (score > 8). The mSEGA comprising Sheet A evaluates frailty on a 13-item scale, which comprises: Medications/Mood/Perception of health/Fall in previous 6 months/Nutrition/Associated diseases/Mobility/Continence/Cognitive function/Age/Place of living/IADL/Meals. Each item is graded either 0 (most favorable state) or 1 or 2 (least favorable state), thus making it possible to classify subjects into three groups: not very frail (score ≤ 8), frail (8 < score ≤ 11), and very frail (score > 11). See details about this scale in Figure 1.

### 2.3. Statistical Analyses

The qualitative variables are presented in absolute values and percentages; the quantitative variables are presented as means and standard deviations. The concordance analysis was completed using Cohen’s kappa test. A kappa coefficient was considered excellent if equal to or greater than 0.81, good between 0.80 and 0.61, average between 0.60 and 0.41, and weak if equal to or less than 0.40. The data were processed using Software R 3.4.0 (*RStudio*, Boston, MA, USA).

### 2.4. Administrative Requirements

All the elderly patients included signed a consent form. As to ethical and regulatory matters, an authorization from the CNIL (Commission nationale de l’informatique et des libertés) was obtained. Registration number: 2099290. The project was in the Ethics Committee under the RCB ID number 2017-A02546-47.

## 3. Results

### 3.1. General Results

One hundred patients over the age of 75 were included during this period. The mean age was 84.34 years, 56 patients (56%) were female, and the average CHARLSON comorbidities score was 4.28 (range: 0–11). The patients’ previous medical histories were remarkable for cardiovascular diseases. The main reason of hospital admission was fall (26 subjects, 26%). Hospitalization was required for 52 subjects (52%). See Table 1.

The details of co-morbidities, the list of usual treatments, and the reasons for the hospitalization of the elderly subjects included during this period are shown in Table 2.

### 3.2. Frailty Assessment

According to the medical assessment of frailty by the mSEGA grid, part A, 73 elderly subjects were considered frail, i.e., 73% of the subjects in the series. The mean mSEGA score is 12.42 (2–24).

The assessment of frailty according to the subjective impressions of the triage nurse, the medical team, and according to the mSEGA score are listed in Table 3.

### 3.3. Concordance Study

Between the medical evaluation of frailty according to the mSEGA grid and the subjective impression of the triage nurse as to the frailty status of the elderly subject upon admission to the emergency department, the agreement, according to Cohen’s kappa, was deemed average.

Between the medical evaluation of frailty according to the mSEGA grid and the subjective impression of the senior physician/interns as to the frailty status of the elderly subject upon admission, the agreement, according to Cohen’s kappa, was deemed good. Between the subjective impression of the senior physician/interns and the subjective impression of the triage nurse as to the frailty status of the elderly subject upon admission, the agreement, according to Cohen’s kappa, was deemed average. See Table 4 for details.

## 4. Discussion

The concept of screening for frailty as a major syndrome has been the subject of many studies since the late 1980s, in particular in the works of Linda Fried, based on sarcopenia [10]. Other works have emphasized the existence of a multidimensional, clinical dimension of frailty, as suggested by Rockwood [11], taking physical, psychological, and social factors into account and providing a frailty index. The SEGA scale, and its modified version developed by the Dramé team [8], takes a multidimensional approach to frailty screening. At present, any score is considered standard throughout general medicine.

The objective of our study was to test the concordance between the feelings of the nursing team, those of the medical team, and the medical use of the mSEGA grid part A on the frailty (or non-frailty) situation of the elderly patient in an emergency department.

The aim is thus to:

1. be able to familiarize all medical and paramedical health professionals with the need for an assessment of frailty in emergencies. This screening would thus be carried out upon admission.
2. create a real “geriatric emergencies” sector (mobile geriatric team strictly dedicated to emergencies or geriatric circuit within the emergency room itself) in order, on the one hand, to optimize the care of elderly subjects and focus on the risk of decompensation of possible geriatric syndromes, and, on the other hand, to reduce the time spent in emergency for these elderly subjects, the number of hospitalizations, as well as their duration.

In our cross-sectional pilot study, we used the SEGA grid multidimensional scale. Our work focused on the use of the mSEGA (part A) frailty grid for elderly subjects over age 75 admitted to the emergency department. Thus, the proportion of frail elderly subjects according to that grid is not insignificant in our study. Furthermore, the completion of this grid is not time-consuming as it takes an estimated 5 minutes on average. It has the advantage of being multidimensional and can be completed by any health care professional working with the elderly subject. We observed an average correlation between the nurse’s subjective opinion of the elderly subject’s “frailty” and the mSEGA grid frailty evaluation according to Cohen’s kappa. The agreement was considered good, according to Cohen’s kappa, between the medical team’s subjective opinion of the elderly subject’s “frailty” and the SEGA grid frailty evaluation. The analysis of the impressions of the health care professionals (triage nurses and doctors) is the main point of our work, which is not found elsewhere in the scientific literature.

This study is novel in that it analyzes the concordance between different health professionals and a frailty evaluation on the mSEGA scale. One of the key features of this study is that it evaluated the concordance between two persons at the time of the frailty evaluation and compared the evaluations of an experienced physician as well as the triage nurse. The scientific literature in this area remains sparse or nearly nonexistent. This agreement, deemed good, between the two professionals shows that frailty evaluation is replicable and reliable even when the evaluator changes. The mSEGA score appears to be a suitable tool for general medicine. This could open the door to establishing consultations specifically geared toward patients’ frailty in order to evaluate whether or not they require assistance by the mobile geriatrics team. Establishing this type of consultation for those over 75 years of age could limit the complications related to underdiagnosed and underestimated frailty. The patient could benefit from greater stability and serenity in remaining at home without limiting everyday activities and senior recreational activities.

Other scales exist, such as the ISAR (Identification of Seniors At Risk) score, developed by a Canadian team led by Jane McCusker in the late 1990s [12]. This is a questionnaire that facilitates the rapid evaluation of a hospitalized elderly person consisting of six targeted yes-or-no questions. Compared with the ISAR, the mSEGA grid is a broader tool, offering a general overview of the patient, taking into consideration the possibility of caregiver burnout. SEGA scale allows setting up a plan for the patient and stimulates contact among the members of the care network. There are also other screening tools for frailty in elderly subjects, such as the TRST (Triage Risk Screening Tool) [13] and the BRIGHT [14] (Brief Risk Identification for Geriatric Health Tool), developed by an American team. The BRIGHT includes 11 items and can be completed by the patient alone or by a loved one. These scores remain in infrequent use.

A limitation of the study is that the study group is small and the data collection period was limited. It would have also been of interest to follow the subjects and see how they were doing (rehospitalizations, falls, etc.) after 1 month and after 6 months. This will be the subject of an additional study that will be carried out in several emergency departments in the Champagne Ardennes region (France) in the coming weeks.

## 5. Conclusions

In order to optimize the care for the elderly subjects over age 75 admitted to the emergency department, screening for frailty remains crucial unless the elderly subject is experiencing a life-threatening acute medical situation. Emergency physicians will, therefore, be aware of the frailty in elderly subjects. The role of a mobile geriatrics unit is crucial in the initial frailty screening conducted in the emergency department (by the triage nurse and/or emergency physicians) in order to optimize intra-hospital care plans and cut down on intra-hospital transfers. It will be useful for emergency medical and paramedical staff to obtain a mSEGA score for any person over the age of 75. The resource person who will determine that score could be the triage nurse. Thus, if the mSEGA score is greater than 8, the mobile geriatrics team will be called upon. This scoring will heighten emergency care teams’ awareness of frailty and geriatric evaluation. Once apprised, based on the degree of urgency of the geriatric evaluation and of the patient’s plan upon leaving the emergency consultation, the mobile geriatrics team will offer an in-department evaluation for hospitalized patients.

## Figures and Tables

**Table 1:** Baseline characteristics of the study population (*n* = 100).

Sex (%)	Female (56%)
Age (mean, range)	84.34 (75–97)
Average Charlson comorbidities score (mean, range)	4.28 (0–11)
Average number of medications (mean, range)	5.71 (0–15)
1e cause of ED admission (%)	Fall (26%)
1e cause of medical history (%)	High blood pressure (74%)
Number of hospitalized patients (%)	52%
Average modified SEGA grid A score (mean, range)	12.42 (1–24)

ED: Emergency Department.

**Table 2:** Characteristics of the study population admitted in emergency unit (*n* = 100).

**Medical History (*n*, %)**	
Arterial hypertension	74 (74%)
Cognitive disorder	21 (21%)
Atrial fibrillation	21 (21%)
Diabetes	14 (14%)
Stroke	12 (12%)
Osteoporosis	12 (12%)
Coronary syndrome	11 (11%)
Phlebitis/pulmonary embolism	11 (11%)
Heart failure	10 (10%)
COPD *	8 (8%)
Chronic renal deficiency	7 (7%)
Prostate cancer	6 (6%)
Epilepsy	5 (5%)
Sleep apnea syndrome	4 (4%)
Colon cancer	3 (3%)
Obliterating arteriopathy of the lower limbs	3 (3%)
Breast cancer	3 (3%)
Pulmonary cancer	2 (2%)
**Treatment (*n*)**	
Antihypertensive	118
Analgesics	54
Proton pump inhibitors	35
Antiplatelet agents	35
Anticoagulants	25
Statins	25
Benzodiazepines	22
Antidepressant	18
Diabetes treatment	17
Vitamin-calcium intakes	14
L-Thyroxin	13
Antipsychotics	8
Alpha blockers	8
Antiepileptics	6
Antidementia	3
**Reason for Admission in Emergency Unit (*n*, %)**	
Cardiovascular	8 (8%)
Pulmonary	15 (15%)
Falls	26 (26%)
Digestive	12 (12%)
Neurological	9 (9%)
Other	30 (30%)

* COPD: Chronic obstructive pulmonary disease.

**Table 3:** Evaluation of frailty.

Triage nurse opinion	65 frail/35 non frail
Medical team opinion	70 frail/30 non frail
Modified SEGA scale (Grid A)	73 frail/ 27 non frail

**Table 4:** Concordance study.

Frailty Evaluation-Concordance Study	Pearson Correlation	Coefficient of Determination (r²)	Cohen’s Kappa
Between the medical evaluation of frailty according to the mSEGA grid and the subjective impression of the triage nurse	0.52	0.27	0.47 (IC95: 0.28–0.67)
Between the medical evaluation of frailty according to the mSEGA grid and the subjective impression of the senior physician	0.76	0.58	0.725 (IC95: 0.57–0.87)
Between the subjective impression of the senior physician/interns and the subjective impression of the triage nurse	0.61	0.37	0.60 (IC95: 0.43–0.77)

## Data Availability

The datasets used and/or analyzed during the current study are available from the corresponding author on reasonable request.
